# Probing Mixed-Genotype Infections II: High Multiplicity in Natural Infections of the Trypanosomatid, *Crithidia bombi,* in Its Host, *Bombus* spp

**DOI:** 10.1371/journal.pone.0049137

**Published:** 2012-11-08

**Authors:** Martina Tognazzo, Regula Schmid-Hempel, Paul Schmid-Hempel

**Affiliations:** Institute of Integrative Biology, ETH Zürich, Zürich, Switzerland; University of Leeds, United Kingdom

## Abstract

Mixed-genotype infections have major consequences for many essential elements of host-parasite interactions. With genetic exchange between co-infecting parasite genotypes increased diversity among parasite offspring and the emergence of novel genotypes from infected hosts is possible. We here investigated mixed- genotype infections using the host, *Bombus* spp. and its trypanosome parasite *Crithidia bombi* as our study case. The natural infections of *C. bombi* were genotyped with a novel method for a representative sample of workers and spring queens in Switzerland. We found that around 60% of all infected hosts showed mixed-genotype infections with an average of 2.47±0.22 (S.E.) and 3.65±1.02 genotypes per worker or queen, respectively. Queens, however, harboured up to 29 different genotypes. Based on the genotypes of co-infecting strains, these could be putatively assigned to either ‘primary’ and ‘derived’ genotypes - the latter resulting from genetic exchange among the primary genotypes. High genetic relatedness among co-infecting derived but not primary genotypes supported this scenario. Co-infection in queens seems to be a major driver for the diversity of genotypes circulating in host populations.

## Introduction

Mixed-genotype infection, that is, the simultaneous presence of more than one genotype of a given parasite species in an infected host individual, is an important and common phenomenon (discussed in e.g. [1], [2]). Mixed-genotype infections have important consequences for many essential elements of host-parasite interactions, such as the dynamics and epidemiology of diseases, or the expression and evolution of virulence - both in theory and by empirical research. For example, mixed-genotype infections of *Plasmodium chabaudi* have been found to be more infective for the mosquito vector than single-genotype infections [3]. Mixed-genotype infections often reach higher parasitaemia for cases as diverse as *Plasmodium mexicanum* infecting lizards [4], microsporidia infecting *Daphnia* (depending on infection pathways) [5], or trypanosomes infecting bumblebees [6]. However, mixed-genotype infections may lead to lower virulence effects on the host depending on the relatedness among co-infecting genotypes [7] and the kind of effects on the host [8]. Indeed, empirical tests show that mixed infections can decrease [9] in virulence when compared to single infections, e.g. [9] although the empirical data is equivocal. Mixed infections of human malaria, for example, associate with a higher probability of clinical symptoms in younger but not older children [10]. In general though, effects of competition between co-infecting parasite genotypes can be observed and may result in the exclusion of some genotypes, depending on the order of infection, such as observed for *P. chabaudi* infecting mice [11], or for anther fungus infecting *Silene* [12]. Most importantly, co-infection by different parasite genotypes also allow for genetic exchange between the co-infecting strains. For the trypanosomatid parasites of humans, for example, this has been shown for different genotypes of *Trypanosoma brucei* during co-infections of the vector (the tsetse fly, *Glossina moritans*) [13], [14], for *Leishmania major* in the sandfly vector (*Phlebotomus dubosqi*) [15], and for *T. cruzi* in experimental co-infections of mammalian cell lines [16]. Such recombination events are known to potentially give raise to new, sometimes persistent and more virulent parasite genotypes as is the case for *Toxoplasma gondii* [17].

Here, we apply a new method to study mixed-genotype infections for field-collected specimens of the intestinal trypanosomatid parasite, *Crithidia bombi* [18], [19] that infects various species of bumblebees (*Bombus* spp.). The study serves to demonstrate the applicability of this method to natural infections, and thereby to illustrate the diversity of parasite genotypes and degree of multiplicity (i.e. the number of different genotypes in a host) that can be found in a representative sample of host individuals from natural populations. The method is described in detail in a companion paper [20] that also demonstrates the feasibility, repeatability and reliability of the procedure.

The *Crithidia - Bombus* system investigated here has been subject to ecological and evolutionary studies for many years. The hosts, *Bombus* spp. (bumblebees), are primitively social insects. In temperate-cool climates, a single queen independently founds a colony in spring. After the first worker brood has hatched, the queen remains in the nest to lay eggs whereas workers forage for nectar and pollen. Over the summer season, the colony grows in worker numbers until sexuals (drones and daughter queens) are produced and leave the nest for mating. Only mated daughter queens hibernate and start their own colony as spring queens the next year. The parasite, *C. bombi*, infects adults of almost all species and castes (queens, workers, and males) of bumblebees. Infection occurs by ingestion of parasite cells with food on flowers [21], or from contaminated nectar stores or brood surfaces in the nest. After several days infective parasite cells are shed with host faeces and can infect a next host upon ingestion. Hence, *C. bombi* is a directly transmitted trypanosomatid and phylogenetically close to *Leishmania* [22]. Since the parasite cannot survive very long outside the host [6] only the hibernating queens can pass on the parasite population to the next year. However, the fitness of infected founding queens (i.e. how many sexual offspring they can produce) is reduced by around 40% [23], and infected workers die earlier when stressed [24].

Surveys of natural host populations in Central Europe have shown that the prevalence of infection obviously varies but is generally rather high (in the order of 30–50% of individuals are infected). From genetic data (i.e. the number of microsatellite alleles at any one locus), around 40–50% of all infected hosts must carry more than one genotype of *C. bombi*. At least two statistical procedures had been applied so far to resolve these mixed-infections into the constituent genotypes [25], [26], but large uncertainties remain and a better insight is needed. Nevertheless, mixed infections of C. *bombi* are highly relevant, since the parasite is known to undergo genetic exchange during co-infections [27]. At the population-wide level, it was estimated that 16.5% of co-infected (and 7% of singly-infected) worker hosts, give rise to new parasite genotypes by genetic exchange (either as recombinants of existing alleles, or likely as a result of slippage giving rise to genotypes with novel alleles). This amounts to approximately 11% of all infected hosts transmitting parasite genotypes different from those that were initially infecting. A substantial number of studies has furthermore shown that infection success (e.g. for bacteria in *Daphnia*, [28] or infection intensity (e.g. for the current system, [6]) depends on which parasite isolate or genotype infects which host genotype. Similarly, the outcome of within-host competition depends on which genotypes infect as, for example, known for trematode infections [29]. Hence, the role of parasite genotype is clearly very important and, therefore, also the characteristics of mixed-genotype infections. Here, we study the situation with field samples that are characteristic for any ecological study.

## Methods

### *C. bombi* Collection and Host Populations

Host collections were done within a larger framework project, of which a sub-sample could be analysed in detail here. Specifically, in the summer of two years (summer 2008, 2009) workers of different species from natural, ecological communities of bumblebees in northern Switzerland were collected. Freshly hibernated spring queens (daughter queens of the previous year) were collected in the following springs at the same locations (spring 2009, 2010). Sampling locations were in the Jura mountains southwest of Basel (Aesch), and in the lowlands east of Zurich (Kartause Ittingen). No specific permits were required for these samplings, nor are the species used here protected. Because genotyping all individuals with our detailed method would have surpassed the then available means, we chose a random sample from among the infected animals to extract the infections for this study, representing around one quarter of all infected hosts. Due to these practical limitations, samples were pooled for an overall assessment of the numerical distribution of infecting genotypes, but not for the estimation of relatedness values where year and caste were analysed separately (see below).

**Figure 1:**
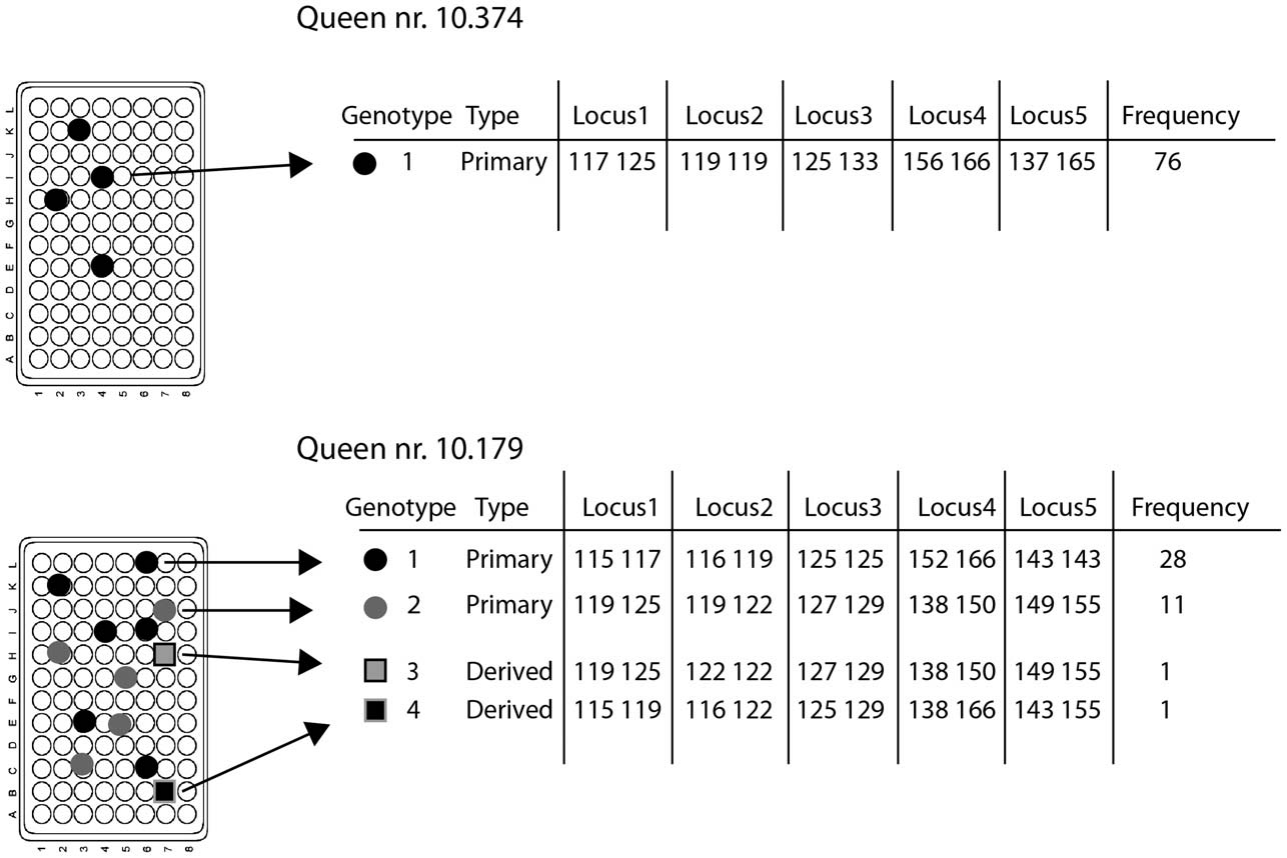
The work protocol. Shown are two examples - **Top panel:** queen nr. 10.374, with a single infection; **Bottom panel:** queen nr. 10.179, with mixed-genotype infection. In each case, the infecting population was separated by FACS into clones and grown in microtiter plates (left). The clones (symbols: circles, squares) were then genotyped by five polymorphic microsatellite loci. Entries are the microsatellite alleles (lengths in bp) observed at each locus (*C. bombi* is diploid). Frequency indicates the number of clones of each type that were found in the infecting population. Note that the allelic pattern of the two “derived” genotypes suggests that nr.3 is a case of allele loss, and nr.4 is a case of recombination as a result of genetic exchange among the two upper genotypes that were considered “primary” due to their higher frequency.

The collected individuals were taken to the laboratory alive where their faeces were checked microscopically for infection with *C. bombi*. Faeces from infected specimens subsequently were processed with the new method described in [20]. In brief, faeces are submitted to single cell sorting, using Fluorescence Activated Cell Sorting (FACS) to start the cloning process. The cells from faeces of each host are directly sorted into 96-well PCR plates (Eppendorf) prefilled with culture medium (see Fig.1). With this method we obtained several single cell clones for most of the infected bees (Table 1; see also Table S1). Each clone was then genotyped (see below).

**Table 1:** Total number of samples collected in the study years.

Year	Caste^a^	Total	Infected	Prevalence^b^
2008	Males	7	0	0
	Queens	18	9	0.500
	Workers	318	100	0.313
2009	Males	24	7	0.294
	Queens	337	18	0.053
	Workers	359	123	0.343
2010	Queens	628	49	0.078
Total		1,691	306	0.181

a Queens in 2008 are daughter queens of that season.

b Prevalence of infections. Castes differ for 2008 (Likelihood ratio = 8.772, *P* = 0.033), and for 2009 (LR = 101.49, *P*<0.001).

### Genotyping and Detection of Mixed-strain Genotype Infections

Genomic DNA was extracted using Direct PCR Lysis for cells (Viagen), following the protocol described in [27]. The multi-locus genotype of clones was characterized using five polymorphic microsatellite markers that have been used repeatedly before [30]. The pattern of microsatellite alleles (alleles are defined by their fragment length in bp) at the five loci defines the multi-locus genotype of an infecting parasite strain. *C. bombi* is genetically diploid (barring the mini- and maxi circles that are characteristic for the kinetoplastids) and the genetic markers used in this study reflect this diploidy. All parasite genotypes found in given individual host bee were typed. For this purpose, 5 µl of the genomic DNA was used in a 20 µl multiplex PCR reaction with primers for the microsatellite loci Cri 4, Cri 2.F10 and Cri 4.G9. A second multiplex PCR for the loci Cri 16 and Cri 1.B6 was run separately (for primer information see [30]). Genotyping was done on a MegaBACE (GE Healthcare) instrument. Analysis of fragments was carried out using the Fragment Profiler software (GE Healthcare).

### Data Analysis

We used zero-truncated Poisson distributions to statistically analyse the number of co-infecting genotypes. Note that the current analysis only refers to *infected* individuals. Due to practical limitations, not every infected individual of the entire sample could be analysed in this study. For this reason, we did not use other plausible statistical approaches such as a zero-inflated Poisson distribution because the remaining samples were only classified as infected/non-infected.

To assess a possible role for putative primary and descending infections in a given host (see Results) we calculated relatedness values between co-infecting genotypes. Relatedness estimates were computed using the Queller-Goodnight estimator [31]. Note that this estimator refers to the genetic background of a given population. We therefore considered the samples collected for a given year and season/caste (i.e., queens in spring 2009, 2010, workers in summer 2008, 2009) as independent populations that defined these backgrounds. The allelic frequencies were correspondingly estimated within that population and used for the estimation of pairwise relatedness between co-infecting parasite genotypes [31]. Relatedness calculations were performed using GenAlEx version 6.4 [32]. Statistical tests were performed in SPSS (PASW Statistics 18.0) for Macintosh and R 2.10.1 for Mac [33]. All P-values refer to two-sided tests, unless mentioned otherwise.

## Results

### Frequency of Mixed-strain Infections

The overall sample sizes and details of collections are given in Table S1. From a total of 1'691 collected individuals over all years and castes, 306 bees were found to be infected by *C. bombi* (18.1% overall prevalence in these samples). Note that these are wild-caught bees of unknown colony origin, being representative samples of the natural populations found in our areas. Our genotypically detailed samples in turn represented around one quarter of all infected hosts, and resulted in a detailed study of the infecting parasites for 82 hosts. In particular, we obtained clones (genotypes derived from single cells) from 45 workers (22 workers in 2008, and 23 in 2009), and from 37 spring queens (15 in 2009 and 22 in 2010; see Table S1). A total of 3,301 clones could successfully be genotyped and these represented a total of 246 different genotypes. Per bee (whose infection is sorted into a 96-well plate) we obtained an average of 39.8±24.2, *n* = 83 plates (s.d.; median: 37) clones (see Table S1). The analysis of clones showed that mixed-genotype infections by *C. bombi* were frequent, with 67% of infected workers and 54% of infected queens carrying mixed-genotype infections, whereas the remainder had only one genotype present (Table 2). On average, we detected 2.47±0.22 (*n* = 37, mean ± s.e.; median: 2) different genotypes per worker, and 3.65±1.03 (*n* = 45, median 2) genotypes per queen.

**Table 2:** Summary statistics of *C. bombi* infections in worker and queen bees over two years (summer 2008 – spring 2010).

Hosts	*N*	Prevalence of mixed-genotype infections^a^	Mean no. different genotypes (± S.E.)^b^	Mean no. primary infections (± S.E.)^c^
Workers	45	0.67	2.47±0.22	2.04±0.18
2008	22	0.59	2.41	2.18
2009	23	0.74	2.52	1.91
Queens	37	0.54	3.65±1.03	1.35±0.10
2009	15	0.53	4.47	1.47
2010	22	0.55	3.09	1.27
Total	82	0.61	3.06	1.71

*N* is number of hosts. Note that the study aim was to characterize a typical sample from the field. Sample sizes are thus too limited to generate a statistic for every host species separately.

a Comparing prevalence of mixed-genotype infections (queens vs. workers): *χ* ^2^ = 1.357, *p* = 0.244.

b Comparing number of different genotypes (queens vs. workers): *t*_80_ = −1.225, *p* = 0.224.

c Comparing number of primary infections (queens vs. workers): *t*_80_ = 3.156, *p* = 0.002.

**Figure 2:**
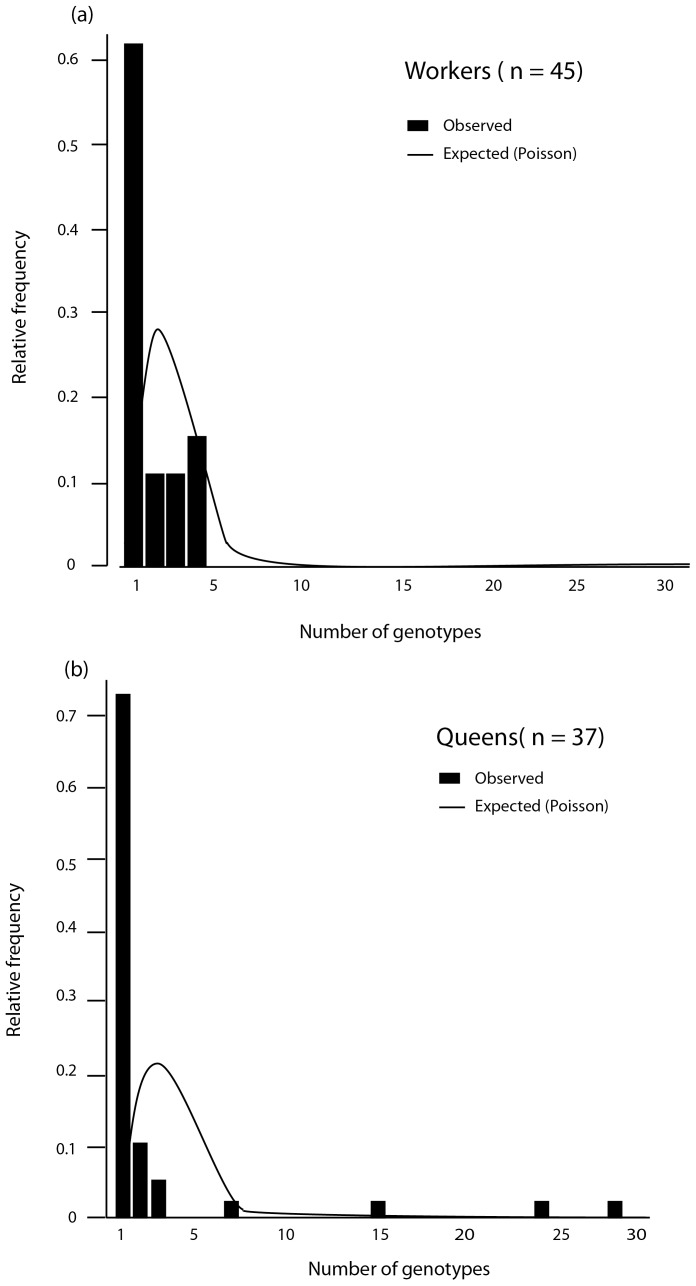
The number of parasite genotypes found in individual hosts. (**a**) Workers have a mean of 2.467±0.22 (S.E. n = 45; black bars) genotypes/host. (**b**) Queens have a mean of 3.65±1.03 (n = 37) genotypes/host. Statistically, the two distributions do not differ from one another, have the same means (glm with quasipoisson: t_81_ = 1.323, p = 0.19), but different variances (see text). Compared to a zero-truncated Poisson expectation (lines), the observed distribution deviates for both castes (Kolmogorov-Smirnov for workers: D = 0.821, p<0.001; for queens: D = 0.714, p<0.001). In the graphs, the first bars refer to multiplicity = 1 (single infections).

Comparing the distributions of the number of genotypes found in queens or workers (Fig. 2), no difference could be found (with Kolomogoroff-Smirnov D = 0.1261, p = 0.9; Permutation test: T = 111, p = 0.27). But in a striking contrast to workers, the queens could also harbour many genotypes, in one case up to 29 (Fig. 2)(with log(1+x)-transformed data: Bartlett-test: K^2^ = 11.126, df = 1, p<0.001). We also checked whether the number of genotypes per host followed a random distribution with a zero-truncated Poisson as the reference. In both groups, there was a significant deviation with too many hosts having fewer genotypes than expected by chance (Fig. 2).

### Primary and Derived Types in Mixed-genotype Infections

In a mixed-genotype infection within a given host we observed that usually one or two genotypes were much more frequent than any other, i.e. the remaining genotypes were generally rare. Furthermore, we noticed that the allele pattern (i.e. the alleles present at any one locus) of the rarer genotypes often were identical or resembled the allele pattern of the frequent genotypes within the same host. This suggested that they could be descendants from these common types. This is plausible, as genetic exchange is known to occur with relatively high frequency between co-infecting genotypes and since our pattern is very similar to that observed in the experimental tests [27]. In our case the descendant, “derived genotypes” could be the product of genetic exchange among the genotypes infecting first (the “primary genotypes”) and which must have resided in the host the longest. According to [27], genetic exchange among two parental (primary) genotypes can lead to derived genotypes via several types of modifications, and residence time seems of the primary infection seems to be of importance for exchange to leave descendants. The modifications include Mendelian recombination, or mutations characterized by allele losses or by having novel alleles at one or more loci (thus, forming novel genotypes). With this hypothesis, all genotypes of a given mixed-genotype infection were putatively assigned as “primary” or “derived” genotype. The relative frequencies of these types are summarized in Table 3. Using this scenario, we also identified the most likely changes that derived genotypes show compared to their respective primary genotypes as follows.

**Table 3:** Fraction of hosts that showed derived genotypes with mutational modifications (alleles lost or gained), or that were recombinants of primary infections.

Caste/Year	*N*	Fraction with mutations	Fraction with recombinants
Workers	45	0.333	0.044
2008	22	0.182	0.045
2009	23	0.478	0.043
Queens	37	0.324	0.135
2009	15	0.200	0.133
2010	22	0.409	0.136
Total	82	0.329	0.073

*N* is the number of investigated host individuals.

#### (a) Alleles lost or gained

A total of 33% of the infected workers and of 32% of the infected queens carried genotypes showing allele losses or gains when the putative derived genotypes were compared to the putative primary genotypes (see Table S2). In other words, compared to the putative primary genotype, the putative derived genotype had a set of different alleles (Table 3, modifications). More specifically, these were: (i) *Allele losses*. In these cases, a certain allele present in the putative primary genotypes is not present in the derived genotypes. In infections of six workers and of five queens, derived genotypes had lost one allele compared to primary genotypes. In workers, none of the genotypes had more than one allele lost. In one queen, the derived genotype lost two alleles. The infection of this one host queen (no. 10.079, Table S2) also contained at least 20 recombinant types. (ii) *Allele gained*. In eight workers and seven queens the putative derived genotypes has a new allele compared to the respective primary genotypes. A gain of two alleles in the derived genotype occurred in two of the infected queens.

#### (b) Recombinants

These are derived genotypes that corresponded to what would result from segregation between the loci of the primary types in a standard meiosis. We thus classified genotypes as ‘recombinant’, when they contained alleles from two different primary genotypes for at least one locus, but otherwise did not show evidence of allele loss or gains (see also [27]). Counting by individual hosts, we found recombinant genotypes in 13.5% of the multiply infected queens, and in 4.4% of the multiply infected workers (Table 3, recombinants; see also Table S2) - a difference that was non-significant (χ^2^_1_ = 2.139, P = 0.14). Counting by the number of genotypes, we found more recombinant genotypes in queens (3.30±1.7, mean ± s.e. genotypes per infection where recombination was inferred) than in workers (0.07±0.05); this difference was marginally significant but only when assuming that queens should *a prior*i have more recombinants as the primary genotypes reside longer in queens than in workers (Mann-Whitney U, one-tailed P = 0.049). Some support comes from the observation that up to 27 different recombinant genotypes could be identified in an individual queen. However, the number of genotypes per host did not correlate with the percentage of recombinant genotypes in the infection (Spearman’s r = 0.162, p = 0.145), suggesting that the diversity of genotypes in a given host was not only determined by the appearance of recombinants. Infected workers had, on average, more putative primary genotypes than infected spring queens (Table 2).

### Relatedness between Parasite Genotypes

Although classifying genotypes within a given host as ‘primary’ or ‘derived’ genotype based on their frequency is somewhat arbitrary, the classification fitted well with he independently assessed overall genetic structure of infections. For this purpose, we calculated the average genetic relatedness, *r*, among pairs of co-infecting genotypes. In particular, we calculated the Queller-Goodnight estimator (a regression estimate based on shared alleles [31] for each population of hosts (i.e. workers 2008, 2009; and queens 2009, 2010, respectively). The estimator gives the probability of gene sharing between ‘individuals’ (our parasite genotypes) beyond the baseline probability set by gene frequencies in the population. Values of *r* significantly greater than zero are considered to show relatedness, whereas negative values result when individuals are less similar than expected from the population mean.

**Figure 3:**
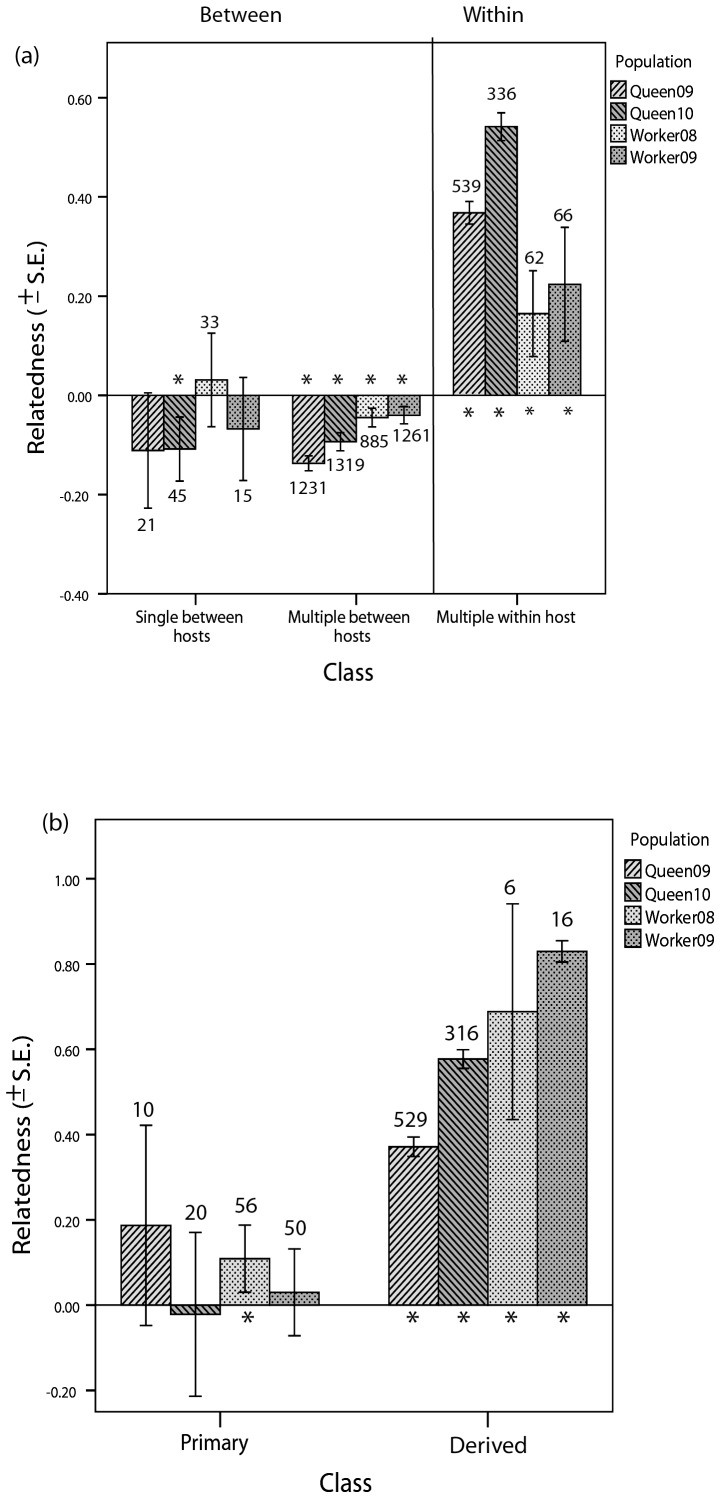
Mean genetic relatedness (Queller-Goodnight estimator) of single and mixed-genotype infections from workers and queens, and in different years. (**a**) Classes comparing single and mixed-genotype infections between and within hosts. Averages of classes are for ‘single between hosts’: *r* = −0.063±0.245 (S.D.); ‘mixed-genotype between hosts’: *r* = −0.081±0.299; ‘mixed-genotype within host’: *r* = 0.405±0.306; (**b**) Co-infecting genotypes within hosts that are classified as either primary or derived. Averages of classes are for ‘primary’: *r* = 0.066±0.348 (S.D.); ‘derived’: *r* = 0.457±0.263. Error bars represent ±1 S.E. Small figures are sample sizes (numbers of pairs). Different shadings represent different castes and years (see legend). Significant deviations from zero at a level of p<0.05 (t-tests for normalized data) for a given class are marked by an asterisk. Populations are the host individuals defining the genotypic background for the relatedness estimator.

Comparing single and mixed-genotype infections between hosts, we found that average relatedness among genotypes was around zero or significantly negative for all populations, i.e. genotypes in different hosts were not particularly close genetically, regardless of whether they originated from single or multiply infected hosts (Fig. 3a). By contrast, the average relatedness among co-infecting genotypes within the same host showed significant positive relatedness values in all populations, with an average *r* = 0.401±0.010 (Fig. 3a). Also the inferred classification into ‘primary’ and ‘derived’ genotypes within the same host was borne out by the relatedness analyses. On average, the average relatedness of primary genotypes was not different from zero, with the exception of population ‘Workers in 2008’ (t = 2.76, df* = *55, p = 0.008). All derived genotypes were, however, significantly related to one another (Fig. 3b).

The *C. bombi* genotypes were further analyzed with respect to the relationships between parasite populations derived from queens and workers, respectively, as they represent the previous and subsequent generations of follow-up years. However, no particular structuring was observed in this case. In other words, the pairwise genetic distances between all populations of genotypes were similar. An analysis of molecular variance (AMOVA) showed that although populations were statistically different from one another (R_ST_ = 0.086, p = 0.01, n = 246 strains), 91% of all molecular variance was contained within or among individuals, and only 9% of the total variance was due to differences among the populations. Hence, at least in this sample, there was no pattern such that infecting genotypes in spring queens were more similar to those circulating in workers of the previous year.

## Discussion

Empirically, mixed-genotype infections are regularly found in a variety of natural host-parasite systems. Examples include such diverse cases as human malaria in East Africa [34], or anther smut in *Silene* in France [35]. In a recent review, Balmer and Tanner [2] found that among 51 human and 21 non-human pathogens an average of 11.3% of infected hosts were carrying mixed-genotype infections. In general, mixed-genotype infections are expected to be more common in high-transmission areas as, for example, observed for *Plasmodium falciparum* (human malaria) [36]. Similarly high values for the prevalence of mixed-genotype infections as in our study are reported, too. Examples are *P. vivax* in several Asian countries (between 30% and 60%; [36]), *P. falciparum* at various locations in Africa (20–30%; [37]), or around 40% for infections by *Trypanosoma cruzi* in Bolivia (20–30%; see also Table 1 in [2]). Our study confirms these patterns and suggests that high prevalence of mixed-genotype infections also pertain to the pollinator community assessed here.

Whereas the prevalence of mixed-genotype infections *per se* can readily be assessed, an estimate of how many genotypes concurrently infect (‘multiplicity’) is much more difficult to achieve. In fact, to resolve an infection into the constituent genotypes from field samples is a major challenge, yet crucial to understand the pattern and dynamics of mixed-genotype infections in nature. Successful previous studies have used a variety of approaches (apart from post-hoc statistical tests, e.g. [25]). For example, isolates from mixed-genotype infections of *Toxoplasma gondii* were grown in cell cultures and the presence of SNP-alleles quantified by pyrosequencing [38]. Mixtures of two different blood parasites (*Plasmodium, Haemoproteus*) in birds were resolved by partially sequencing a gene (cytochrome b) and assessing the occurrence of double peaks in the electropherograms [39]; similar methods were used in a study of *T. gondii* in chickens [40]. From previous pilot studies, however, we know that such estimates from electropherograms are not reliable in the case of *C. bombi* infecting bumblebee hosts. Therefore, we have here used direct cloning from single cells of mixed-genotype infections using cell sorting as the first step (see companion paper; [20]). This allowed the assessment of the number, distribution, and identity of genotypes of an infecting population in unprecedented detail (Fig. 2). Our detailed values again compare with values reported in other studies. For example, Bogreau and co-workers [37] (malaria in humans) estimate a rather low average multiplicity of around 1.2, whereas Lopez-Villavicencio and co-workers [35] find an average of 2.33 genotypes of parasitic fungi per host plant, again similar to the estimate here; Cui and colleagues [36] suggest up to six co-infecting genotypes for *P. vivax* in New Guinea. Although such numbers are hardly comparable in a strict sense, and all methods are unable to detect very rare genotypes, the comparison suggests that our observed multiplicities of around 2.5 in workers and 3.7 in queens, respectively, are in good, upper range. This is emphasized by the maximum number of 29 co-infecting genotypes in one of the queens (see Table S2).

We here have putatively classified the co-infecting genotypes into primary and descendants. This is obviously a tentative classification. Nevertheless, we emphasize that the classification is based on data from experimental studies on genetic exchange among co-infecting *C. bombi* [27]. As a word of caution, our classification is based on the relative frequencies of co-infecting genotypes. From previous studies on the growth of infecting *C. bombi* [41], [42] and experimentally-staged mixed-genotype infections [43], it is indeed very likely that the most common genotypes in a given host are the ones that have infected first. However, this need not necessarily be the case [44]. Nevertheless, our count and genetic data are compatible with the following putative scenario. Becoming infected in spring (or when having picked up an infection before hibernation), the young queens will sometimes carry more than one genotype (in about half of the cases, see Table 3). Given the life cycle of queens (that live for one year) *vs.* workers (that live for around 3–4 weeks), infections in queens can reside longer than in workers. As a result, genetic exchange among co-infecting genotypes (the ‘primary genotypes’) is thereby facilitated and results in an array of derived genotypes that are present in the same host individual. Some of these descendant genotypes will be recombinants of the primary genotypes, and some will be mutants (alleles lost or gained). These genotypes can then be passed on to a next host. We thus suggest that genetic exchange among co-infecting *C. bombi*, primarily in spring queens, is important for the emergence of novel types in this system.

Similar considerations - although at different scales - have been made for *Toxoplasma gondii* [45], [46], or *Plasmodium falciparum* [47] where sexual cycles, generating genetic diversity, exist. In *Trypanosoma cruzi* (Chagas disease; [48] three hybridization events appear responsible for the current worldwide population structure. But obviously, the emergence of new genotypes by genetic exchange is not the only force that can act in our system. Instead, a given host environment will select for certain kinds of genotypes rather than others. Such specificity has been observed in this system numerous times before - for example, in the studies of [6], [49], or [30]. Experiments have also shown the action of selection, as mixed-genotype infections typically become filtered out depending on host colony, such that only a subset of the primary infection cocktail will infect and persist [43], [50]. Moreover, selection and a process of filtering among co-infecting genotypes relate to host defences, as supported by experimental work [51]. A final element of selection could occur when the co-infecting genotypes would compete amongst each other. Competition is tentatively suggested by the observation that multiplicity seems skewed towards fewer co-infecting genotypes as expected by chance (Fig. 2).

Recently, it was found that the probability of infecting daughter queens inside the colony is correlated with number of genotypes circulating in the worker bees [43]. Therefore high genotype diversity in the bumblebee colony makes it more likely that at least one of the genotypes could infect the next-year generation of hosts. Furthermore, the relatively high levels of novel genotypes emerging in multiply infected bees (Table 3) means that a small number of hosts (those prone to mixed-genotype infections) could contribute to a disproportionate share of the parasite genotypes in a population. In fact, in experimental tests some colonies appear to be resistant to most genotypes of the parasite, whereas other colonies are susceptible to almost any genotype [6], [30], [41], [43], [49]. This suggests that susceptible hosts are a major source of new infections for other hosts and can thus probably be viewed as super-spreaders, which are considered to be the main drivers of an epidemic (e.g. [52].

## Supporting Information

Table S1 **Overview of** ***C. bombi*** **samples.** Bee no. indicates the running index of a given host individual, collected in a given year an belonging to a case (worker/queen).
(DOCX)

Table S2 **Recombination between primary genotypes to generate the observed derived genotypes.** Only cases of mixed-genotype infections are listed. In all cases shown here, recombination was inferred (for classification, see text). The putative recombinant genotypes derived from the two primary genotypes are labelled as Reco 1, etc.). Additionally, in the host specimens no. 10.079 and no. 10.179 mutations of the primary genotypes (i.e. allele loss, allele gain, see text) were inferred. The entries are the alleles (fragment length in bp) of a given genotype at the five loci (Cri 2.F10 to Cri 1.B6) used in this study. Clone frequency is the number of clones that were retrieved for a given genotype and in a given host (e.g. from host no. N09.139 a total of 53 clones could be isolated and they are classified into 3 genotypes).
(DOCX)
