# Supplementary material for: Probing Mixed-Genotype Infections II: High Multiplicity in Natural Infections of the Trypanosomatid, Crithidia bombi, in Its Host, Bombus spp
Source: PLoS One. 2012 Nov 8;7(11):e49137. doi: 10.1371/journal.pone.0049137 (PMC3493493; doi:10.1371/journal.pone.0049137)
Supplement: Table S1 — Overview of C. bombi samples. Bee no. indicates the running index of a given host individual, collected in a given year an belonging to a case (worker/queen). (DOCX) [file pone.0049137.s001.docx]

**Table S1**

| Host  (Bee no.) | Year | Caste | No. clones  analysed^a^ | Distinct genotypes^b^ | Primary  infections^c^ | Genotype change^d^ | |
| --- | --- | --- | --- | --- | --- | --- | --- |
| BJ08.002 | 2008 | worker | 36 | 2 | 2 |  |  |
| BJ08.003 | 2008 | worker | 7 | 1 | 1 |  |  |
| BJ08.006 | 2008 | worker | 44 | 4 | 3 | m |  |
| BJ08.008 | 2008 | worker | 2 | 1 | 1 |  |  |
| BJ08.009 | 2008 | worker | 12 | 2 | 2 |  |  |
| BJ08.065 | 2008 | worker | 38 | 2 | 2 |  |  |
| BJ08.073 | 2008 | worker | 13 | 2 | 2 |  |  |
| BJ08.085 | 2008 | worker | 32 | 4 | 3 | m |  |
| BJ08.087 | 2008 | worker | 29 | 5 | 5 |  |  |
| BJ08.088 | 2008 | worker | 27 | 4 | 4 |  |  |
| BJ08.165 | 2008 | worker | 33 | 1 | 1 |  |  |
| BJ08.169 | 2008 | worker | 16 | 2 | 1 | m |  |
| BJ08.174 | 2008 | worker | 44 | 4 | 4 |  |  |
| BJ08.177 | 2008 | worker | 23 | 5 | 4 | m |  |
| BJ08.179 | 2008 | worker | 1 | 1 | 1 |  |  |
| BJ08.182 | 2008 | worker | 21 | 5 | 4 | r |  |
| BJ08.183 | 2008 | worker | 29 | 1 | 1 |  |  |
| BJ08.185 | 2008 | worker | 5 | 1 | 1 |  |  |
| BJ08.187 | 2008 | worker | 38 | 1 | 1 |  |  |
| BJ08.191 | 2008 | worker | 52 | 3 | 3 |  |  |
| BJ08.320 | 2008 | worker | 10 | 1 | 1 |  |  |
| BJ08.322 | 2008 | worker | 5 | 1 | 1 |  |  |
| Total | 2008 | workers | 517 | 53 |  |  |  |
| BJ09.237 | 2009 | worker | 31 | 1 | 1 |  |  |
| BJ09.253 | 2009 | worker | 56 | 1 | 1 |  |  |
| BJ09.264 | 2009 | worker | 15 | 2 | 1 | m |  |
| BJ09.305 | 2009 | worker | 18 | 5 | 4 | m |  |
| BJ09.306 | 2009 | worker | 38 | 3 | 3 |  |  |
| BJ09.320 | 2009 | worker | 50 | 5 | 3 | m |  |
| BJ09.322 | 2009 | worker | 30 | 2 | 2 |  |  |
| BJ09.329 | 2009 | worker | 14 | 1 | 1 |  |  |
| BJ09.330 | 2009 | worker | 37 | 3 | 2 | m |  |
| BJ09.383 | 2009 | worker | 39 | 5 | 3 | m |  |
| BJ09.394 | 2009 | worker | 3 | 2 | 1 | m |  |
| BJ09.406 | 2009 | worker | 41 | 1 | 1 |  |  |
| BJ09.422 | 2009 | worker | 27 | 4 | 4 |  |  |
| BJ09.428 | 2009 | worker | 54 | 2 | 1 | m |  |
| BJ09.446 | 2009 | worker | 26 | 5 | 4 | m |  |
| BJ09.452 | 2009 | worker | 11 | 1 | 1 |  |  |
| BJ09.458 | 2009 | worker | 2 | 2 | 1 | m |  |
| N09.139 | 2009 | worker | 54 | 3 | 2 | r |  |
| N09.157 | 2009 | worker | 13 | 2 | 2 |  |  |
| N09.160 | 2009 | worker | 10 | 2 | 2 |  |  |
| N09.175 | 2009 | worker | 24 | 2 | 1 | m |  |
| N09.180 | 2009 | worker | 2 | 1 | 1 |  |  |
| N09.192 | 2009 | worker | 27 | 3 | 2 | m |  |
| Total | 2009 | workers | 622 | 58 |  |  |  |
| 09.032 | 2009 | queen | 16 | 1 | 1 |  |  |
| 09.049 | 2009 | queen | 57 | 2 | 2 |  |  |
| 09.064 | 2009 | queen | 81 | 29 | 2 | r |  |
| 09.065 | 2009 | queen | 68 | 1 | 1 |  |  |
| 09.066 | 2009 | queen | 47 | 4 | 4 |  |  |
| 09.152 | 2009 | queen | 33 | 1 | 1 |  |  |
| 09.159 | 2009 | queen | 26 | 1 | 1 |  |  |
| 09.174 | 2009 | queen | 55 | 3 | 1 | m |  |
| 09.181 | 2009 | queen | 89 | 2 | 2 |  |  |
| 09.187 | 2009 | queen | 47 | 1 | 1 |  |  |
| 09.221 | 2009 | queen | 60 | 2 | 1 | m |  |
| 09.285 | 2009 | queen | 15 | 1 | 1 |  |  |
| 09.287 | 2009 | queen | 61 | 16 | 2 | r |  |
| 09.318 | 2009 | queen | 66 | 2 | 1 | m |  |
| BJ09.087 | 2009 | queen | 34 | 1 | 1 |  |  |
| Total | 2009 | queens | 755 | 67 |  |  |  |
| 10.005 | 2010 | queen | 62 | 2 | 1 | m |  |
| 10.027 | 2010 | queen | 23 | 1 | 1 |  |  |
| 10.035 | 2010 | queen | 26 | 3 | 2 | r |  |
| 10.079 | 2010 | queen ^e^ | 135 | 25 | 2 | r/m |  |
| 10.127 | 2010 | queen | 68 | 1 | 1 |  |  |
| 10.130 | 2010 | queen | 59 | 1 | 1 |  |  |
| 10.132 | 2010 | queen | 86 | 4 | 2 | m |  |
| 10.133 | 2010 | queen | 27 | 2 | 1 | m |  |
| 10.159 | 2010 | queen | 78 | 1 | 1 |  |  |
| 10.179 | 2010 | queen | 45 | 8 | 2 | r/m |  |
| 10.187 | 2010 | queen | 37 | 1 | 1 |  |  |
| 10.189 | 2010 | queen | 46 | 2 | 2 |  |  |
| 10.207 | 2010 | queen | 74 | 1 | 1 |  |  |
| 10.211 | 2010 | queen | 73 | 1 | 1 |  |  |
| 10.290 | 2010 | queen | 61 | 2 | 1 | m |  |
| 10.361 | 2010 | queen | 82 | 2 | 1 | m |  |
| 10.374 | 2010 | queen | 76 | 1 | 1 |  |  |
| 10.439 | 2010 | queen | 81 | 1 | 1 |  |  |
| 10.441 | 2010 | queen | 50 | 1 | 1 |  |  |
| 10.486 | 2010 | queen | 58 | 3 | 2 | m |  |
| 10.493 | 2010 | queen | 87 | 3 | 1 |  |  |
| 10.520 | 2010 | queen | 73 | 2 | 1 | m |  |
| Total | 2010 | queens | 1,407 | 68 |  |  |  |
|  |  |  |  |  |  |  |  |
| Gross total |  | All | 3,301 | 246 |  |  |  |

^a^ Number of clones from this host that could be genotyped.

^b^ Number of different genotypes of *C. bombi* in this host.

^c^ Inferred number of primary, independent infecting genotypes in this host.

^d^ Inferred change for derived genotypes in this host: mutation (m), recombinant (r). See text for details. Missing values: some genotypes could not be unequivocally assigned.

^e^ For this individual, two 96-well plates were used for sorting the faeces (one plate in all other cases).
