# Supplementary material for: Probing Mixed-Genotype Infections II: High Multiplicity in Natural Infections of the Trypanosomatid, Crithidia bombi, in Its Host, Bombus spp
Source: PLoS One. 2012 Nov 8;7(11):e49137. doi: 10.1371/journal.pone.0049137 (PMC3493493; doi:10.1371/journal.pone.0049137)
Supplement: Table S2 — Recombination between primary genotypes to generate the observed derived genotypes. Only cases of mixed-genotype infections are listed. In all cases shown here, recombination was inferred (for classification, see text). The putative recombinant genotypes derived from the two primary genotypes are labelled as Reco 1, etc.). Additionally, in the host specimens no. 10.079 and no. 10.179 mutations of the primary genotypes (i.e. allele loss, allele gain, see text) were inferred. The entries are the alleles (fragment length in bp) of a given genotype at the five loci (Cri 2.F10 to Cri 1.B6) used in this study. Clone frequency is the number of clones that were retrieved for a given genotype and in a given host (e.g. from host no. N09.139 a total of 53 clones could be isolated and they are classified into 3 genotypes). (DOCX) [file pone.0049137.s002.docx]

**Table S2**

| Hosts | Strain type | Cri 2.F10 | Cri 16 | Cri 4 | Cri 4.G9 | Cri 1.B6 | Clone frequency |
| --- | --- | --- | --- | --- | --- | --- | --- |
|  |  |  |  |  |  |  |  |
| Workers 2008 | |  |  |  |  |  |  |
| BJ08.182 | Primary A | 117 | 116 119 | 129 131 | 154 | 137 149 | 2 |
|  | Primary B | 117 125 | 110 119 | 125 129 | 138 150 | 137 145 | 7 |
|  | Reco 1 | 117 125 | 116 119 | 125 131 | 138 154 | 137 149 | 1 |
|  |  |  |  |  |  |  |  |
| Workers 2009 | |  |  |  |  |  |  |
| N09.139 | Primary A | 117 125 | 116 119 | 129 131 | 150 156 | 143 165 | 48 |
|  | Primary B | 117 125 | 116 119 | 125 | 150 156 | 143 165 | 4 |
|  | Reco 1 | 117 125 | 116 119 | 125 131 | - | 143 165 | 1 |
|  |  |  |  |  |  |  |  |
| Queens 2009 | |  |  |  |  |  |  |
| 09.064 | Primary A | 117 | 119 | 125 133 | 150 154 | 151 153 | 4 |
|  | Primary B | 117 125 | 119 | 125 129 | 158 | 137 143 | 3 |
|  | Reco 1 | 117 | 119 | 125 133 | 154 158 | 143 153 | 3 |
|  | Reco 2 | 117 | 119 | 125 | 154 158 | 137 153 | 2 |
|  | Reco 3 | 117 125 | 119 | 129 133 | 154 158 | 143 151 | 2 |
|  | Reco 4 | 117 | 119 | 125 133 | 150 158 | 143 151 | 2 |
|  | Reco 5 | 117 | 119 | 129 133 | 150 158 | 137 151 | 2 |
|  | Reco 6 | 117 125 | 119 | 125 | 154 158 | 137 153 | 2 |
|  | Reco 7 | 117 125 | 119 | 125 129 | 150 158 | 143 153 | 2 |
|  | Reco 8 | 117 125 | 119 | 125 | 154 158 | 143 153 | 2 |
|  | Reco 9 | 117 125 | 119 | 129 133 | 150 158 | 143 153 | 2 |
|  | Reco 10 | 117 | 119 | 125 | 154 158 | 137 151 | 1 |
|  | Reco 11 | 117 | 119 | 125 | 150 158 | 137 151 | 1 |
|  | Reco 12 | 117 | 119 | 125 | 154 158 | 143 153 | 1 |
|  | Reco 13 | 117 125 | 119 | 125 | 154 158 | 143 151 | 1 |
|  | Reco 14 | 117 125 | 119 | 125 | 154 158 | 137 151 | 1 |
|  | Reco 15 | 117 125 | 119 | 125 | 150 158 | 137 153 | 1 |
|  | Reco 16 | 117 | 119 | 125 129 | 150 158 | 143 151 | 1 |
|  | Reco 17 | 117 | 119 | 125 129 | 154 158 | 143 151 | 1 |
|  | Reco 18 | 117 | 119 | 125 129 | 150 158 | 143 153 | 1 |
|  | Reco 19 | 117 | 119 | 125 133 | 154 158 | 137 153 | 1 |
|  | Reco 20 | 117 | 119 | 125 133 | 154 158 | 137 151 | 1 |
|  | Reco 21 | 117 125 | 119 | 125 133 | 154 158 | 143 151 | 1 |
|  | Reco 22 | 117 | 119 | 129 133 | 154 158 | 143 153 | 1 |
|  | Reco 23 | 117 | 119 | 129 133 | 154 158 | 143 151 | 1 |
|  | Reco 24 | 117 | 119 | 129 133 | 150 158 | 137 153 | 1 |
|  | Reco 25 | 117 125 | 119 | 129 133 | 154 158 | 143 153 | 1 |
|  | Reco 26 | 117 125 | 119 | 129 133 | 154 158 | 137 151 | 1 |
|  | Reco 27 | 117 125 | 119 | 129 133 | 150 158 | 137 153 | 1 |
|  |  |  |  |  |  |  |  |
| 09.287 | Primary A | 117 125 | 119 122 | 125 129 | 138 144 | 137 153 | 41 |
|  | Primary B | 117 125 | 116 119 | 129 133 | 138 156 | 137 143 | 6 |
|  | Reco 1 | 117 | 119 122 | 129 | 138 144 | 137 | 1 |
|  | Reco 2 | 117 125 | 116 119 | 129 | 138 144 | 143 153 | 1 |
|  | Reco 3 | 117 | 119 122 | 129 | 138 | 137 143 | 1 |
|  | Reco 4 | 117 125 | 116 119 | 129 | 138 144 | 137 153 | 1 |
|  | Reco 5 | 117 | 116 119 | 129 | 138 144 | 137 | 1 |
|  | Reco 6 | 117 | 116 122 | 129 | 144 156 | 143 153 | 1 |
|  | Reco 7 | 125 | 116 122 | 129 | 144 156 | 137 153 | 1 |
|  | Reco 8 | 117 | 119 | 125 129 | 144 156 | 137 143 | 1 |
|  | Reco 9 | 117 | 116 122 | 125 133 | 144 156 | 143 153 | 1 |
|  | Reco 10 | 117 125 | 119 | 125 133 | 138 156 | 137 153 | 1 |
|  | Reco 11 | 117 125 | 119 122 | 125 133 | 138 156 | 137 153 | 1 |
|  | Reco 12 | 117 125 | 119 122 | 129 133 | 144 156 | 137 143 | 1 |
|  | Reco 13 | 117 125 | 119 122 | 129 133 | 138 | 143 153 | 1 |
|  | Reco 14 | 117 | 116 119 | 129 133 | 138 144 | 137 143 | 1 |
|  |  |  |  |  |  |  |  |
| Queens 2010 | |  |  |  |  |  |  |
| 10.035 | Primary A | 117 125 | 119 | 129 131 | 148 150 | 137 149 | 9 |
|  | Primary B | 117 | 119 | 129 131 | 138 156 | 143 | 16 |
|  | Reco 1 | 117 | 119 | 129 131 | 150 156 | 143 149 | 1 |
|  |  |  |  |  |  |  |  |
| 10.079 | Primary A | 117 | 119 | 129 131 | 152 154 | 143 155 | 59 |
|  | Allele gain | 117 | 116 119 | 129 131 | 152 154 | 143 155 | 1 |
|  | Primary B | 117 | 119 | 129 | 144 154 | 137 153 | 44 |
|  | Allele loss | 117 | 119 | 129 | 144 | 137 153 | 2 |
|  | Allele loss | 117 | 119 | 129 | 154 | 153 | 1 |
|  | Reco 1 | 117 | 119 | 129 131 | 144 154 | 137 143 | 1 |
|  | Reco 2 | 117 | 119 | 129 131 | 144 154 | 137 155 | 1 |
|  | Reco 3 | 117 | 119 | 129 131 | 144 154 | 153 155 | 1 |
|  | Reco 4 | 117 | 119 | 129 131 | 152 154 | 137 143 | 2 |
|  | Reco 5 | 117 | 119 | 129 131 | 152 154 | 137 155 | 3 |
|  | Reco 6 | 117 | 119 | 129 131 | 144 152 | 137 143 | 1 |
|  | Reco 7 | 117 | 119 | 129 131 | 154 | 153 155 | 1 |
|  | Reco 8 | 117 | 119 | 129 131 | 154 | 137 155 | 1 |
|  | Reco 9 | 117 | 119 | 129 | 154 | 137 143 | 2 |
|  | Reco 10 | 117 | 119 | 129 | 154 | 153 155 | 1 |
|  | Reco 11 | 117 | 119 | 129 | 144 152 | 137 155 | 3 |
|  | Reco 12 | 117 | 119 | 129 | 144 154 | 143 153 | 3 |
|  | Reco 13 | 117 | 119 | 129 | 144 152 | 153 155 | 1 |
|  | Reco 14 | 117 | 119 | 129 | 144 152 | 143 153 | 1 |
|  | Reco 15 | 117 | 119 | 129 | 144 154 | 153 155 | 1 |
|  | Reco 16 | 117 | 119 | 129 | 144 154 | 137 143 | 1 |
|  | Reco 17 | 117 | 119 | 129 | 144 154 | 137 155 | 1 |
|  | Reco 18 | 117 | 119 | 129 | 152 154 | 143 153 | 1 |
|  | Reco 19 | 117 | 119 | 129 | 152 154 | 137 143 | 1 |
|  | Reco 20 | 117 | 119 | 129 | 152 154 | 137 155 | 1 |
|  |  |  |  |  |  |  |  |
| 10.179 | Primary A | 115 117 | 116 119 | 125 | 152 166 | 143 | 28 |
|  | Allele loss | 115 117 | 119 | 125 | 152 166 | - | 1 |
|  | Primary B | 119 125 | 119 122 | 127 129 | 138 150 | 149 155 | 11 |
|  | Allele loss | 119 125 | 122 | 127 129 | 138 150 | 149 155 | 1 |
|  | Reco 1 | 117 125 | 119 122 | 125 129 | 138 166 | 143 149 | 1 |
|  | Reco 2 | 115 125 | 119 122 | 125 129 | 150 152 | 143 149 | 1 |
|  | Reco 3 | 115 125 | 116 122 | 125 129 | 138 152 | 143 149 | 1 |
|  | Reco 4 | 115 119 | 116 122 | 125 129 | 138 166 | 143 155 | 1 |
